# Appalachia: Putting the “Critical” in Race and Crowdsourcing a Pathway Model on Institutional Racism

**DOI:** 10.13023/jah.0303.01

**Published:** 2021-07-25

**Authors:** Lauri Andress, Keri Valentine

**Affiliations:** West Virginia University; West Virginia University

**Keywords:** Appalachia, race, commentary, pathway model, equity, racism, institutional, social determinants of health

## Abstract

As the website *Understanding and Dismantling Racism: Crowdsourcing a Pathway Model in Appalachia* explains, we are seeking assistance in refining a pathway model that elucidates institutional racism from the unique standpoint of Appalachia. We think that Appalachia has a distinctive cultural toolkit that shapes its orientation on issues. Our goal is to use crowdsourcing to harness this unique Appalachian ethos to refine the Pathway model on Institutional Racism based on comments, edits, questions, and ideas left on the website.

Note: This commentary is linked to a review of the website, which appears in this same issue under Media Reviews and Reports.

Hudson M. Review of: Pathway Model IDC WVU Faculty Senate. Understanding and Dismantling Racism: Crowdsourcing a Pathway Model in Appalachia. J Appalach Health 2021;3(3):124–9. DOI: https://doi.org/10.13023/jah.0303.11

## INTRODUCTION

As the website Understanding and Dismantling Racism: Crowdsourcing a Pathway Model in Appalachia^1^ explains, we are seeking assistance in refining a pathway model that highlights institutional racism from the unique standpoint of Appalachia. The need for such a model manifested from social justice efforts to address racism and racial injustice at West Virginia University and the larger community. Specifically, we sought to disrupt the individual-level “hearts and minds” work that overtakes efforts to address racism without contextualizing the historical and present-day systems and structures that undergirds racism and its impacts.

The goal of this website is to use crowdsourcing to refine this model based on comments, edits, questions, and ideas left on the website. For immediate participation, with no further background, you may skip this commendatory and provide input on the model. https://forms.gle/uV4AJdrzMuLWJah56. Alternatively, we invite you to read on for suppositions behind this endeavor.

## THE SPECIFICNESS OF APPALACHIA

What is the “there” of Appalachia that might shape a set of beliefs and ideals around race and racism? This exercise asserts that Appalachia represents both a position on a map as well as an ideologic position that defines the region’s way of thinking about phenomena.^2^–^6^ We learned early on that the most effective and accurate way to portray the social determinants of health (SDOH) relies on a human geography maxim that spaces are only places by virtue of those who inhabit that space imprinting it with the attendant cultural toolkit.^7^ This cultural toolkit, which operates in any given region, is the silent engine that shapes the many acts of “being” in relation to a space so that the space becomes a place imbued with social processes, beliefs, ideals, and values.

This duality of Appalachia as a region on a map plus a way of being is supported by countless geographic displays, legislative declarations, data sets, narratives, and mission statements. As such, we think that Appalachia has a distinctive cultural toolkit that shapes its orientation on issues.

## APPLYING A CRITICAL RACE LENS AND INSTITUTIONAL RACISM

A critical-lens approach to race and racism asks us to elevate the associated sociologic, historical economic, and political investigation from the individual unit of analysis to an institutional level.^8^,^9^ In this case, we consider how racism and discrimination are embedded in societal structures and systems operating through social, institutional, organizational, and governmental processes, procedures, policies, and practices. (N.B. Discrimination is used in this case to represent other forms of stigmatization and loss of opportunities and resources based on social class that may stem from religion, sexuality, or income level). Anytime you have multiple systems and outcomes that act across time to regularly and consistently disadvantage one group while privileging another, an ethical and responsible society must ask and examine why this is so.

Consider outcomes that steadfastly, indiscriminately, and disproportionately distribute harms to one group in the areas of multiple systems including health, housing, education, criminal justice, education, wealth, and income.^10^,^11^ A critical lens applied to racism and race facilitates an examination of the legacy of slavery in the U.S. Although legal slavery ended with the U.S. Civil War and the passage of the 13th Amendment to the Constitution, our country continues to struggle with this history.^12^

Inequality, lack of economic opportunity, and poor health persist today broadly in American society, defined both by social class and by race and ethnicity. Finally, to those who would equate seeing race to racism while asserting the merits of colorblindness we would say, “not so” and what is more, in contrast, we assert the harms of a colorblind society. As one scholar argues, colorblindness may still result in the same outcomes as overt forms of racism: maintenance of white privilege.^13^

Whiteness benefits whites. Whites receive material gains by adhering to racial ideologies that keep them on top. Thus, finding ways to reproduce and maintain a white position of privilege becomes paramount, and in the present-day U.S., that means espousing the language of colorblindness. To put it more simply, how can it be ethical to flourish in a society devoid of interrogation when one part of that society consistently experiences the daily harm of oppressive policies and institutions? As responsible citizens and scientists, it is our obligation to address structural racism and discrimination that strike at the very heart of our Nation and its stability.

## WHY A PATHWAY MODEL?

Efforts to address the SDOH to achieve health equity encompass multilevel approaches that work at the macro and micro levels across different sectors of society using a variety of tools. Pathway models are one such tool that schematically displays a vision of how society can trace out the route for these complex, multi-tiered and many sectored interactions that work over potentially long time periods. As a pathway model, this depiction of how institutional racism functions is a powerful tool, capable of illustrating the often-omitted socially constructed systems, policies, and institutions that serve as visible gatekeepers to resources and opportunities for groups that experience racism. In other words, pathway models are suitable to a critical lens approach to racism because it is able to portray how social and biological causes work in tandem in cases where our understanding tends to be less well developed.

The task with which a pathway model excels is mapping the social and biological processes and the interaction between them in order to develop an explanation of how the biological systems in the human body change in ways that are determined by social as well as biological/biochemical processes. This is at the heart of the intellectual challenge of the social determination of health and the corresponding inequities in health. With these pathway models we deliver more refined tools that allow us to study and design policies, systems and rules that reflect the differential contextual stimuli and their respective interactive chains that shape the molecules in the human body according to the social position someone is assigned.

## CONCLUSION

As conscientious, principled citizens and scientists, we should consider it our charge to address how structural racism and discrimination affect the causal pathways to well-being defined not only as health but also as the ability to thrive and assume lives of equality as we define it in the U.S. Whether because you believe in a cultural toolkit that has established an ethos of Appalachia, or because you are concerned about the Nation and the impact of institutional racism and discrimination, or you have studied the social determinants of health and considered the kind of evidence needed to analyze nonmedical determinants of health (maybe all of these), please visit the website https://sites.google.com/view/idc-pathway-model/home and help in refining a pathway model that explains institutional racism from the unique standpoint of Appalachia.
